# Timing of surgery and outcomes in patients with congenital pulmonary airway malformation: a national inpatient database study

**DOI:** 10.1007/s00383-025-06188-3

**Published:** 2025-09-08

**Authors:** Naohiro Takamoto, Shotaro Aso, Takaaki Konishi, Michimasa Fujiogi, Kaori Morita, Mai Kutsukake, Yoshitsugu Yanagida, Hiroki Matsui, Kiyohide Fushimi, Hideo Yasunaga, Jun Fujishiro

**Affiliations:** 1 Department of Pediatric Surgery, Graduate School of Medicine, The University of Tokyo, 7-3-1 Hongo, Bunkyo-ku, Tokyo, Japan; 2 Department of Clinical Epidemiology and Health Economics, School of Public Health, The University of Tokyo, 7-3-1 Hongo, Bunkyo-ku, Tokyo, Japan; 3 Department of Health Services Research, Graduate School of Medicine, The University of Tokyo, 7-3-1 Hongo, Bunkyo-ku, Tokyo, Japan; 4 Department of Health Policy and Informatics, Institute of Science Tokyo, 1-5-45 Yushima, Bunkyo-ku, Tokyo, 113-8510 Japan

**Keywords:** Bacterial pneumonia, Congenital pulmonary airway malformation, Elective surgical procedure, Symptomatic, Thoracoscopy

## Abstract

**Purpose:**

The timing of elective surgery for asymptomatic congenital pulmonary airway malformation (CPAM) at birth remains controversial. We aimed to describe characteristics and outcomes of patients who underwent surgery for CPAM.

**Methods:**

We retrospectively identified patients aged < 18 years who were hospitalized for CPAM during the neonatal period and underwent surgery between July 2010 and March 2022 using the Diagnosis Procedure Combination database in Japan. We grouped eligible patients into those aged < 28 days (neonatal group) and ≥ 28 days (infant group) at surgery. Outcomes included in-hospital mortality, morbidity, duration of anesthesia, and hospital stay.

**Results:**

We identified 105 neonates and 287 infants (including 89, 107, and 91 aged 28 days to 5 months, 6–12 months, and > 12 months, respectively). In-hospital mortality and morbidity were similar among the groups. In the infant group, duration of anesthesia was longer in those with preoperative admission due to bacterial pneumonia or who underwent thoracoscopic surgery. Infants with congenital malformation or emergency admission had longer hospital stays.

**Conclusions:**

In-hospital mortality and morbidity were comparable among the different timings of surgery for CPAM. Preoperative bacterial pneumonia and thoracoscopic surgery could be risk factors for long duration of anesthesia but not for long hospital stay.

**Supplementary Information:**

The online version contains supplementary material available at 10.1007/s00383-025-06188-3.

## Introduction

Congenital pulmonary airway malformation (CPAM) is a pulmonary abnormality that results from aberrant lung development during fetal life [1]. The incidence of CPAM is 1 in 10,000–35,000 births [2]. At birth, approximately one-quarter of patients with CPAM are symptomatic [3]. For patients with symptoms of respiratory distress at birth, early surgical resection of the lesions is recommended [4, 5].

Between 36 and 97% of patients with asymptomatic CPAM at birth can remain asymptomatic during childhood [6]. Because patients with CPAM lesions are likely to contract pneumonia, surgical resection of the lesions can become hard due to adhesion formation [4]. In addition, early resection can contribute to compensatory lung growth [4]. Thus, elective surgical resection of the lesions is performed during childhood in case of prophylactic surgery for patients with asymptomatic CPAM [4].

However, the optimal timing of surgery for asymptomatic CPAM at birth remains controversial [7, 8]. Generally, surgery for asymptomatic CPAM is performed during the first year of life because symptoms are likely to develop at 5–10 months of age [3, 5] and initial postnatal development of the lungs takes place between birth and 1–2 years of age [4]. A questionnaire survey of pediatric surgeons from 48 countries reported that 62% of surgeries for asymptomatic CPAM were performed at 6–12 months of age [9]. In contrast, several previous studies recommended early surgery at 1–6 months of age to avoid infection and to encourage compensatory lung growth [10, 11].

Therefore, the aim of the present study was to describe characteristics and outcomes of patients who underwent surgery for CPAM classified by age at surgery, and to identify the optimal timing and other factors affecting the outcomes of surgery for asymptomatic CPAM, using a national inpatient database.

## Methods

### Database

This retrospective cohort study used the Diagnosis Procedure Combination database, which collects the data of approximately 8,000,000 inpatients from more than 1200 hospitals each year. All 82 academic hospitals are required to participate in the database, whereas participation by community hospitals is optional [12]. Fifty percent of the hospitals certified by the Japan Surgical Society are included in the database. Moreover, 80% of hospitals with pediatric intensive care units and 90% of hospitals with neonatal intensive care units are included in this database [13, 14].

The Diagnosis Procedure Combination database collects data comprising unique hospital identifiers, sex, age, body weight, main diagnoses, comorbidities at admission, complications after admission, ambulance use, emergency or elective admission, surgeries and procedures, duration of anesthesia, length of stay, and total cost of hospitalization. The diagnosis of disease is defined as textual data from the Japanese and International Classification of Diseases, Tenth Revision (ICD-10) codes. Previous studies indicated that this database had a high validity of the recorded diagnoses, procedures, and operative information [15, 16]. This database has been used in several studies on pediatric and thoracic surgery [17, 18].

The present study was approved by our institutional review board (approval number 3501-(5), dated May 19, 2021). The requirement for informed consent was waived because the patient data were anonymized.

### Study protocol

We retrospectively identified patients aged < 18 years who were hospitalized for congenital cystic lung (ICD-10 code: Q33.0) during the neonatal period between July 2010 and March 2022. We excluded patients (i) who were diagnosed with bronchial atresia (Q32.3, Q32.4, J98.0, and P25.0), bronchopulmonary sequestration (Q33.2), or foregut duplication cysts (Q34.1 and J98.4) and (ii) who did not undergo surgery for lung lesions. Because J98.4 originally included congenital cystic lung, we differentiated them by checking the Japanese texts.

First, the neonatal group was divided from the eligible patients because most of the patients with symptomatic CPAM underwent surgery during the neonatal period in previous studies [5, 19]. Second, we named the residual eligible patients as the infant group and further divided them into 28 days to 5 months, 6–12 months, and > 12 months because previous questionnaire surveys about the timing of surgery for asymptomatic CPAM in Canada and Europe used < 6 months and > 12 months as the cutoffs [9, 20].

We described the demographic and clinical characteristics, including sex, age, and body weight at surgery, premature birth, congenital malformation except for CPAM, respiratory support within the first 2 days after admission during the neonatal period, emergency admission, ambulance use, hospital volume, preoperative admission due to bacterial pneumonia (ICD-10 codes: A48.1, J10.0, J11.0, J12, J13, J14, J15, J16, J17.0, J17.8, J18, J85, and J86) [21, 22], video-assisted thoracoscopic surgery, and surgical procedures.

The primary outcome was in-hospital mortality. The secondary outcomes were morbidity, reoperation, perioperative blood transfusion and catecholamines use, admission to intensive care unit, length of stay in the intensive care unit, 30-day readmission due to CPAM, duration of anesthesia, length of stay, and total hospitalization costs. In-hospital morbidity included surgical site infection (ICD-10 codes: T79.3, T81.3, T81.4, and T94.1), postoperative bleeding (T81.0, T81.1), respiratory complications (J12–18, J69.0, J95.8, J95.9, and J96.0), pulmonary embolism (I26), cardiac event (I21–I24, I50), stroke (I60–I64), urinary tract infection (N10, N12, N30, and N39.0), acute renal failure (N17), and sepsis (A40, A41). We defined the currency exchange rate as 120 Japanese yen per 1 US dollar.

To focus on patients with asymptomatic CPAM at birth, we performed multivariable regression analyses for duration of anesthesia and length of stay in the infant group, adjusting for patient and hospital characteristics. The covariates included sex, age, and body weight at surgery, congenital malformation, emergency admission, hospital volume, preoperative admission due to bacterial pneumonia, and thoracoscopic surgery.

We performed a subgroup analysis for patients who underwent thoracoscopic surgery in the infant group to reveal outcomes in different age groups.

We performed a sensitivity analysis for patients in the infant group who did not need respiratory support during the neonatal period because patients who received respiratory support during the neonatal period may be symptomatic.

We used the Fisher’s exact test to analyze binary and categorical variables and the Kruskal–Wallis test to analyze continuous variables. A two-sided significance level of 0.05 was used to test each hypothesis. We conducted all statistical analyses using Stata/SE 18.0 (Stata Corp. LLC, College Station, TX, USA).

## Results

**Fig. 1:**
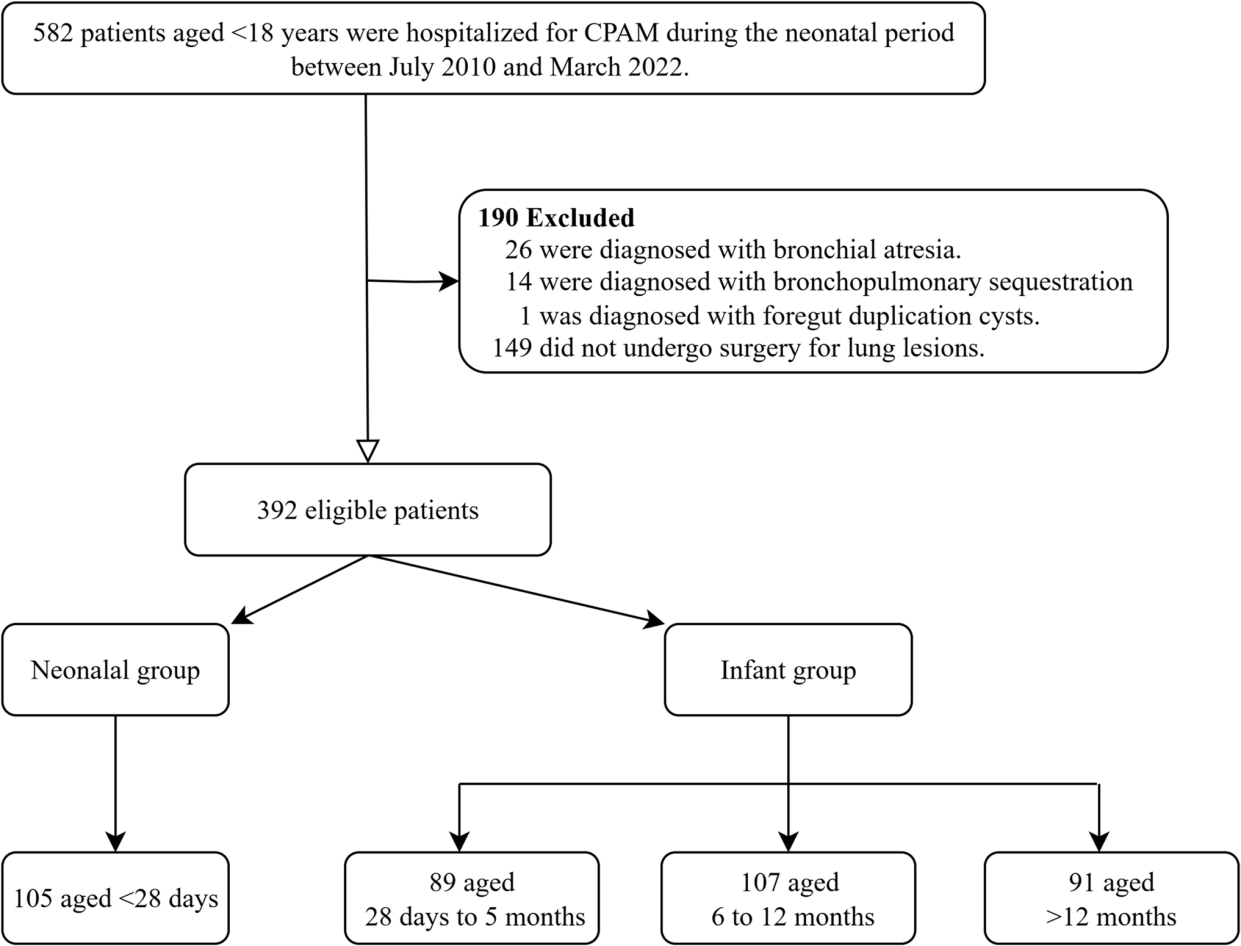
Patient flow diagram

Figure 1 shows the flow chart. A total of 582 patients aged < 18 years were hospitalized for CPAM during the neonatal period between July 2010 and March 2022. Of these patients, 190 patients were excluded: (i) 26 patients, 14 patients, and 1 patient were diagnosed with bronchial atresia, bronchopulmonary sequestration, and foregut duplication cysts, respectively; and (ii) 149 patients did not undergo surgery for lung lesions (4 neonates and 1 infant aged 51 days died without undergoing surgery). Thus, a total of 392 patients were eligible, including 105 aged < 28 days, 89 aged 28 days to 5 months, 107 aged 6–12 months, and 91 aged > 12 months.

Table 1 shows the demographic and clinical characteristics of all patients. In total, the proportion of males and premature births was 60% and 12%, respectively. Neonates comprised 27% of the total patients. The proportions of respiratory support and emergency admission were the largest in the neonatal group. Approximately half of the neonatal group underwent mechanical ventilation. The proportion of preoperative admissions due to bacterial pneumonia differed between the groups. Lobectomy was performed in 94% of the patients. The proportion of thoracoscopic surgery was the largest in patients aged > 12 months.

**Table 1:** Demographic and clinical characteristics of all patients, categorized by age

	Neonatal group				Infant group				*P*-value*
	< 28 days		28 days–5 months		6–12 months		> 12 months		
	*n* = 105		*n* = 89		*n* = 107		*n* = 91		
Male sex	66	(63)	45	(51)	64	(60)	54	(59)	0.36
Age at surgery, days	5	(1–16)	111	(68–147)	249	(218–309)	505	(418–833)	< 0.001
Body weight at admission, kg	3.0	(2.7–3.3)	5.9	(4.3–6.8)	8.0	(7.1–8.6)	9.8	(8.8–11.0)	< 0.001
Premature birth (< 37 weeks)	16	(15)	9	(10)	12	(11)	12	(13)	0.72
Congenital malformation	11	(10)	11	(12)	11	(10)	9	(9.9)	0.96
Respiratory support^†^	70	(67)	18	(20)	21	(20)	24	(26)	< 0.001
Oxygen	20	(19)	8	(8.9)	10	(9.3)	12	(13)	0.13
Nasal high flow	1	(1.0)	0	(0.0)	0	(0.0)	0	(0.0)	0.73
IPPV	2	(1.9)	0	(0.0)	3	(2.8)	0	(0.0)	0.18
Mechanical ventilation	50	(48)	10	(11)	9	(8.4)	13	(14)	< 0.001
Emergency admission	67	(64)	15	(17)	1	(0.9)	1	(1.1)	< 0.001
Ambulance use	5	(4.8)	1	(1.1)	0	(0.0)	0	(0.0)	0.001
Hospital volume, per year									0.005
≤ 1.6	44	(42)	39	(44)	45	(42)	58	(64)	
> 1.6	61	(58)	50	(56)	62	(58)	33	(36)	
Preoperative admission^‡^	0	(0.0)	3	(3.4)	6	(5.6)	7	(7.7)	0.016
Number of times admitted before surgery^‡^									0.006
1	0	(0.0)	3	(3.4)	3	(2.8)	7	(7.7)	
2	0	(0.0)	0	(0.0)	3	(2.8)	0	(0.0)	
Thoracoscopic surgery	16	(15)	27	(30)	27	(25)	50	(55)	< 0.001
Surgical procedure									< 0.001
Open wedge resection	3	(2.8)	1	(1.1)	2	(1.9)	1	(1.1)	
Open lobectomy	86	(82)	60	(67)	78	(73)	39	(43)	
Thoracoscopic wedge resection	2	(1.9)	3	(3.4)	2	(1.9)	8	(8.8)	
Thoracoscopic lobectomy	14	(13)	25	(28)	25	(23)	43	(47)	

Data are presented as *n* (%) or median (interquartile range)

*IPPV* intermittent positive pressure ventilation

^*^The *P*-value was calculated to compare four groups using Fisher’s exact test for binary and categorical variables, and the Kruskal–Wallis test for continuous variables

^†^Respiratory support within the first 2 days after admission during the neonatal period

^‡^Preoperative admission due to bacterial pneumonia

Table 2 shows the comparison of the outcomes of all patients. In-hospital mortality and morbidity, reoperation, and 30-day readmission due to CPAM did not differ among the four groups. The neonatal group had the highest proportion of blood transfusion and catecholamine use, the longest length of stay in the intensive care unit, the shortest duration of anesthesia, the longest length of stay, and the highest total hospitalization cost among the four groups.

**Table 2:** Comparisons of outcomes of all patients, categorized by age

	Neonatal group				Infant group				*P*-value*
	< 28 days		28 days–5 months		6–12 months		> 12 months		
	*n* = 105		*n* = 89		*n* = 107		*n* = 91		
In-hospital mortality	2	(1.9)	0	(0.0)	1	(0.9)	0	(0.0)	0.57
In-hospital morbidity^†^	9	(8.6)	11	(15)	11	(10)	5	(5.5)	0.42
Surgical site infection	1	(1.0)	0	(0.0)	2	(1.9)	2	(2.2)	0.69
Postoperative bleeding	4	(3.8)	4	(4.5)	4	(3.7)	1	(1.1)	0.59
Respiratory complication	7	(6.7)	6	(6.7)	6	(5.6)	2	(2.2)	0.44
Pulmonary embolism	0	(0.0)	1	(1.1)	0	(0.0)	0	(0.0)	0.23
Cardiac events	0	(0.0)	1	(1.1)	0	(0.0)	0	(0.0)	0.23
Sepsis	1	(1.0)	1	(1.1)	0	(0.0)	1	(1.1)	0.71
Reoperation	2	(1.9)	1	(1.1)	2	(1.9)	1	(1.1)	0.99
Blood transfusion	47	(45)	20	(22)	12	(11)	8	(8.8)	< 0.001
Catecholamine use	63	(60)	15	(17)	9	(8.4)	8	(8.8)	< 0.001
ICU admission	92	(88)	58	(65)	70	(65)	54	(59)	< 0.001
After surgery	86	(82)	50	(56)	70	(65)	54	(59)	< 0.001
Duration of ICU stay									< 0.001
≤ 2 days	4	(3.8)	40	(45)	52	(49)	49	(54)	
3–7 days	7	(6.7)	7	(8.0)	18	(17)	5	(5.5)	
> 7 days	81	(77)	11	(13)	0	(0.0)	0	(0.0)	
30-day readmission^‡^	0	(0.0)	1	(1.1)	0	(0.0)	0	(0.0)	0.23

	Median	IQR	Median	IQR	Median	IQR	Median	IQR	
Duration of anesthesia, minutes	252	(200–310)	305	(259–339)	334	(275–390)	331	(281–413)	< 0.001
Length of stay, days	38	(25–58)	10	(8–15)	10	(8–11)	9	(8–11)	< 0.001
Total hospitalization cost, USD	47,753	(39,308–64,318)	17,617	(15,910–19,929)	17,418	(16,279–18,645)	16,605	(15,654–18,604)	< 0.001

Data are presented as *n* (%) or median (interquartile range)

*ICU* intensive care unit, *IQR* interquartile range, *USD* United States dollars

^*^The *P*-value was calculated to compare four groups using Fisher’s exact test for binary and categorical variables, and the Kruskal–Wallis test for continuous variables

^†^The items “urinary tract infection” and “acute renal failure” were omitted from in-hospital morbidities because no patients presented with these items

^‡^Readmission due to congenital pulmonary airway malformation

Table 3 shows the results of the multivariable regression analysis for duration of anesthesia in the infant group. Longer duration of anesthesia was significantly associated with preoperative admission due to bacterial pneumonia [coefficient, 64.6 min; 95% confidence interval (CI) 16.6–112.6; *P* = 0.008] and thoracoscopic surgery (coefficient, 30.1 min; 95% CI 5.8–54.6; *P* = 0.015). The timing of surgery for CPAM was not associated with the duration of anesthesia.

**Table 3:** Multivariable regression analysis for duration of anesthesia in the infant group

	Coef	95% CI	*P*-value
Sex			
Male	15.3	− 7.0 to 37.6	0.18
Female	Ref		
Age at surgery			
28 days–5 months	Ref		
6–12 months	25.5	− 4.8 to 55.7	0.098
> 12 months	38.0	− 0.9 to 77.0	0.056
Body weight at surgery, kg	− 1.0	− 6.9 to 4.8	0.73
Congenital malformation			
Yes	6.7	− 29.1 to 42.4	0.71
No	Ref		
Emergency admission			
Yes	− 15.8	− 67.5 to 35.9	0.55
No	Ref		
Hospital volume, per year			
≤ 1.6	Ref		
> 1.6	− 9.6	− 32.4 to 12.9	0.40
Preoperative admission*			
Yes	64.6	16.6–112.6	0.008
No	Ref		
Thoracoscopic surgery			
Yes	30.1	5.8–54.6	0.015
No	Ref		

*Coef* coefficient, *CI* confidence interval, *Ref* reference

^*^Preoperative admission due to bacterial pneumonia

Table 4 shows the results of the multivariable regression analysis for length of stay in the infant group. Longer hospital stay was significantly associated with congenital malformation (coefficient, 9.0 days; 95% CI 1.6–16.3; *P* = 0.017) and emergency admission (coefficient, 61.6 days; 95% CI 50.9–72.2; *P* < 0.001). The timing of surgery for CPAM was not associated with the length of stay.

**Table 4:** Multivariable regression analysis for length of stay in the infant group

	Coef	95% CI	*P*-value
Sex			
Male	− 0.9	− 4.7 to 4.5	0.97
Female	Ref		
Age at surgery			
28 days–5 months	Ref		
6–12 months	− 1.3	− 7.5 to 4.9	0.68
> 12 months	0.3	− 7.7to 8.2	0.95
Body weight at surgery, kg	− 0.8	− 2.0 to 0.4	0.20
Congenital malformation			
Yes	9.0	1.6–16.3	0.017
No	Ref		
Emergency admission			
Yes	61.6	50.9–72.2	< 0.001
No	Ref		
Hospital volume, per year			
≤ 1.6	Ref		
> 1.6	− 1.1	− 5.7 to 3.6	0.65
Preoperative admission*			
Yes	− 7.7	− 17.5 to 2.2	0.13
No	Ref		
Thoracoscopic surgery			
Yes	− 0.3	− 5.3 to 4.7	0.90
No	Ref		

*Coef* coefficient, *CI* confidence interval, *Ref* reference

^*^Preoperative admission due to bacterial pneumonia

Supplemental Tables 1, 2, 3 and 4 show the results of the sensitivity analysis for the infant group patients who did not need respiratory support during the neonatal period. Among the infant group, the largest proportion of emergency admissions was in patients aged 28 days to 5 months. Also, among the infant group, patients aged 28 days to 5 months had the highest proportion of blood transfusion use, the longest length of stay in the intensive care unit, and the shortest duration of anesthesia. Preoperative admission due to bacterial pneumonia was significantly associated with a longer duration of anesthesia (coefficient, 177.9 min; 95% CI 75.0–280.9; *P* = 0.001). Emergency admission was significantly associated with longer length of hospital stay (coefficient, 128.9 days; 95% CI 91.7–166.1; *P* < 0.001). The timing of surgery for CPAM was not associated with duration of anesthesia or length of hospital stay.

Supplemental Tables 5, 6, 7 and 8 show the results of the subgroup analysis for patients in the infant group who underwent thoracoscopic surgery. Among the infant group, the proportions of emergency admissions and high-volume hospitals were the largest in patients aged 28 days to 5 months. Additionally, patients aged 28 days to 5 months had the longest length of stay in the intensive care unit and the shortest duration of anesthesia. Patients aged > 12 months were significantly associated with a longer duration of anesthesia (coefficient, 117.5 min; 95% CI 39.2–195.7; *P* = 0.004). Emergency admission was significantly associated with a longer length of hospital stay (coefficient, 16.3 days; 95% CI 11.3–21.2; *P* < 0.001).

## Discussion

The present study reviewed the demographic and clinical characteristics and outcomes of patients who underwent surgery for CPAM classified by age at surgery, using a national database in Japan. The neonatal group showed higher proportions of respiratory support, emergency admission, blood transfusion, and catecholamine use, and longer stay in the intensive care unit than the infant group; however, the neonatal group had the shortest duration of anesthesia among the groups. Patients older than 12 months at the time of surgery showed the highest prevalence of preoperative admission due to bacterial pneumonia and the highest proportion of thoracoscopic surgery among the four groups. No significant differences were observed in in-hospital mortality and morbidity among the groups. In the infant group, preoperative admission due to bacterial pneumonia and thoracoscopic surgery were associated with longer durations of anesthesia, whereas congenital malformation and emergency admission were associated with longer hospital stays. The sensitivity analysis showed that preoperative admission due to bacterial pneumonia and emergency admission were associated with longer durations of anesthesia and longer hospital stays, respectively.

In the present study, patients in the neonatal group likely had long intensive care stays. Physiological anemia and hemodynamic instability commonly occur during the neonatal period [23, 24]. Because surgery can substantially impact hemodynamics in neonates, the neonatal group could have a higher proportion of blood transfusion and catecholamine use and longer stays in the intensive care unit than the infant group. Furthermore, the proportions of patients requiring respiratory support and emergency admission in the neonatal group were higher than those in the infant group. This finding is likely because patients with symptomatic CPAM at birth generally require respiratory support and emergency admission due to respiratory distress [5]. Indeed, the present proportion of the neonatal group (27%) was consistent with the proportion of patients with symptomatic CPAM at birth reported previously [3].

Although the findings of the present study revealed a need for intensive care among neonates, in-hospital mortality and morbidity did not differ across the different age groups. In previous studies, in-hospital mortality and morbidity were 0–7.5% and 4.2–28%, respectively [5, 7, 19, 25]. These results were comparable with those in the present study. To our knowledge, the present study was the first study to compare the mortality and morbidity between neonates and infants. The equivalence of these outcomes for both neonates and infants may be attributed to the exclusion of neonates who died before surgery due to poor conditions. These results indicate that neonates can undergo surgery as safely as infants.

In the present study, only one-third of the patients underwent surgery at 6–12 months of age, and the proportion of patients aged > 12 months was larger than that of the previous studies [9, 20]. Additionally, the proportion of thoracoscopic surgery in patients aged > 12 months was significantly higher than that in patients aged 6–12 months (55% vs. 25%). The possible reason is that some pediatric surgeons could have waited until the patient reached 12 months of age for thoracoscopic surgery [26, 27] because a bronchial blocker, which is preferred for pediatric thoracoscopic surgery, is indicated for patients aged > 12 months [28, 29]. Although the present study showed that patients aged > 12 months were more likely to have prolonged duration of anesthesia, thoracoscopic surgery after the age of 12 months may be acceptable because long-term pulmonary function following thoracoscopic lobectomy was better than that following open lobectomy and the timing of lobectomy did not impact the long-term pulmonary function [30, 31].

Preoperative admission due to bacterial pneumonia was significantly associated with longer duration of anesthesia. CPAM can lead to respiratory symptoms such as fever, dyspnea, and respiratory distress [3, 5]. Previous studies reported that the median age of developing symptomatic CPAM was 6.9–7.5 months and the incidence increased with long follow-up periods: 3.2% (range 2.5–7.5 months) and 24% (range 3 months to 6 years)[3, 5]. For asymptomatic CPAM, preoperative pulmonary infection is one of the important factors in selecting the timing of surgery because extensive fibrotic adhesions can affect the difficulty of thoracic surgery [4, 5, 26, 32, 33]. Besides, long operative time for CPAM was related to old age, recurrent respiratory infection, and thoracoscopic surgery in previous studies [7, 33–36]. Similarly, based on the findings of the main and sensitivity analyses in the present study, preoperative admission due to bacterial pneumonia can be a risk factor for the long duration of anesthesia.

Unlike previous studies showing the association of long hospital stays with recurrent respiratory infection and open surgery, in the main and sensitivity analyses of the present study, a longer hospital stay was associated with emergency admission; the timing of elective surgery was not a risk factor for either outcome. Even if a patient undergoes surgery at an older age and has had episodes of previous infection, their hospital stay may not increase despite the longer operative time due to the difficulty of operation. Information on these risks could be useful when explaining perioperative courses to patients and their parents.

The present study had several limitations. First, we did not have information on the severity of the bacterial pneumonia: vital signs, symptoms, laboratory data (e.g., white blood cells), or imaging findings (e.g., computed tomography scans). Second, the number of patients hospitalized for bacterial pneumonia before surgery may have been underestimated because we were unable to count the number of patients who were admitted to other hospitals for such treatment. Third, we were unable to differentiate emergency from elective surgery and thus assessed emergency admission as a proxy of emergency surgery. Fourth, we were unable to obtain detailed operative data. Conversion from thoracoscopic to open surgery could not be determined due to the nature of the database. We may have overestimated the duration of anesthesia and length of stay in the thoracoscopic surgery group, even though conversion seldom occurs (2.3–15% of thoracoscopic surgery) [7, 34, 36]. We were also unable to obtain data on operative time. Instead, we assessed the duration of anesthesia. According to a validation study regarding the current database, the duration of anesthesia was approximately 1 h longer than operative time [16]. Fifth, we did not perform multivariable regression analyses for in-hospital morbidity and adverse events because of the small sample size. Sixth, our study did not present patients with CPAM who did not receive surgery. We were unable to follow up with patients across different hospitals in this database. A hospital where a patient was diagnosed with CPAM was possibly different from one where the patient received surgery for CPAM. If we included patients who did not receive surgery, the same patients were doubly identified. Finally, we were unable to examine long-term postoperative pulmonary function. Although early lobectomy at 1–6 months of age for compensatory lung growth had been proposed [10, 11], those who underwent lobectomy for CPAM at approximately 1 year of age had a compatible pulmonary function with healthy people [30, 37].

## Conclusions

The timing of surgery for CPAM was not associated with in-hospital mortality or morbidity. In patients with asymptomatic CPAM, the risk factors for a long duration of anesthesia and hospital stay could be preoperative admission due to bacterial pneumonia and emergency admission, respectively.

## Supplementary Information

Below is the link to the electronic supplementary material.

Supplementary file1 (DOCX 267 KB)

## Data Availability

This study used the Diagnosis Procedure Combination database.
