# Supplementary material for: Timing of surgery and outcomes in patients with congenital pulmonary airway malformation: a national inpatient database study
Source: Pediatr Surg Int. 2025 Sep 8;41(1):288. doi: 10.1007/s00383-025-06188-3 (PMC12417296; doi:10.1007/s00383-025-06188-3)
Supplement: Supplementary file 1 — Supplementary file1 (DOCX 267 KB) [file 383_2025_6188_MOESM1_ESM.docx]

**Supplemental Table 1. Demographic and clinical characteristics of patients who did not need respiratory support during the neonatal period in the infant group, categorized by age**

|  |  | Infant group | | | | | | | |  |  |
| --- | --- | --- | --- | --- | --- | --- | --- | --- | --- | --- | --- |
|  |  | 28 days–5 months | |  | 6–12 months | |  | >12 months | |  |  |
|  |  | n=71 | |  | n=86 | |  | n=67 | |  | P-value* |
| Male sex |  | 35 | (49) |  | 50 | (58) |  | 39 | (58) |  | 0.47 |
| Age at surgery, days |  | 118 | (78–147) |  | 251 | (218–312) |  | 504 | (395–847) |  | <0.001 |
| Body weight at admission, kg |  | 6.0 | (4.8–6.9) |  | 8.0 | (7.2–8.7) |  | 9.7 | (8.8–11.2) |  | <0.001 |
| Premature birth (< 37 weeks) |  | 4 | (5.6) |  | 7 | (8.1) |  | 7 | (10) |  | 0.59 |
| Congenital malformation |  | 7 | (9.9) |  | 6 | (7.0) |  | 7 | (10) |  | 0.70 |
| Emergency admission |  | 8 | (11) |  | 1 | (1.2) |  | 1 | (1.5) |  | 0.005 |
| Ambulance use |  | 0 | (0.0) |  | 0 | (0.0) |  | 0 | (0.0) |  |  |
| Hospital volume, per year |  |  |  |  |  |  |  |  |  |  | 0.090 |
| ≤1.6 |  | 30 | (42) |  | 36 | (42) |  | 39 | (58) |  |  |
| >1.6 |  | 41 | (58) |  | 50 | (58) |  | 28 | (42) |  |  |
| Preoperative admission^†^ |  | 2 | (2.8) |  | 4 | (4.7) |  | 6 | (9.0) |  | 0.29 |
| Number of times admitted before surgery^†^ |  |  |  |  |  |  |  |  |  |  | 0.12 |
| '1 |  | 2 | (2.8) |  | 2 | (2.3) |  | 6 | (9.0) |  |  |
| '2 |  | 0 | (0.0) |  | 2 | (2.3) |  | 0 | (0.0) |  |  |
| Thoracoscopic surgery |  | 21 | (30) |  | 21 | (24) |  | 39 | (58) |  | <0.001 |
| Surgical procedure |  |  |  |  |  |  |  |  |  |  | <0.001 |
| Open wedge resection |  | 0 | (0.0) |  | 2 | (2.3) |  | 1 | (1.5) |  |  |
| Open lobectomy |  | 50 | (70) |  | 63 | (73) |  | 26 | (39) |  |  |
| Thoracoscopic wedge resection |  | 2 | (2.8) |  | 2 | (2.3) |  | 7 | (10) |  |  |
| Thoracoscopic lobectomy |  | 19 | (27) |  | 19 | (22) |  | 33 | (49) |  |  |

Data are presented as n (%) or median (interquartile range).

* The P-value was calculated to compare four groups using Fisher’s exact test for binary and categorical variables, and the Kruskal–Wallis test for continuous variables.

^†^ Preoperative admission due to bacterial pneumonia.

Abbreviation: IPPV, intermittent positive pressure ventilation

**Supplemental Table 2. Comparisons of outcomes of patients who did not need respiratory support during the neonatal period in the infant group,** **categorized by age**

|  | Infant group | | | | | | | |  |  |
| --- | --- | --- | --- | --- | --- | --- | --- | --- | --- | --- |
|  | 28 days–5 months | |  | 6–12 months | |  | >12 months | |  |  |
|  | n=71 | |  | n=86 | |  | n=67 | |  | P-value* |
| In-hospital mortality | 0 | (0.0) |  | 1 | (1.2) |  | 0 | (0.0) |  | 0.99 |
| In-hospital morbidity† | 8 | (11) |  | 10 | (12) |  | 2 | (3.0) |  | 0.10 |
| Surgical site infection | 0 | (0.0) |  | 2 | (2.3) |  | 1 | (1.5) |  | 0.64 |
| Postoperative bleeding | 3 | (4.2) |  | 3 | (3.5) |  | 0 | (0.0) |  | 0.32 |
| Respiratory complication | 4 | (5.6) |  | 6 | (7.0) |  | 0 | (0.0) |  | 0.072 |
| Pulmonary embolism | 1 | (1.4) |  | 0 | (0.0) |  | 0 | (0.0) |  | 0.62 |
| Cardiac events | 1 | (1.4) |  | 0 | (0.0) |  | 0 | (0.0) |  | 0.62 |
| Sepsis | 0 | (0.0) |  | 0 | (0.0) |  | 1 | (1.5) |  | 0.30 |
| Reoperation | 0 | (0.0) |  | 2 | (2.3) |  | 1 | (1.5) |  | 0.64 |
| Blood transfusion use | 15 | (21) |  | 9 | (10) |  | 5 | (7.5) |  | 0.046 |
| Catecholamine use | 9 | (13) |  | 5 | (5.8) |  | 6 | (9.0) |  | 0.34 |
| ICU admission | 41 | (58) |  | 55 | (64) |  | 38 | (57) |  | 0.61 |
| After surgery | 38 | (54) |  | 55 | (64) |  | 38 | (57) |  | 0.38 |
| Duration of ICU stay |  |  |  |  |  |  |  |  |  | 0.004 |
| ≤2 days | 31 | (44) |  | 39 | (45.3) |  | 35 | (52) |  |  |
| 3–7 days | 6 | (8.5) |  | 16 | (18.6) |  | 3 | (4.5) |  |  |
| >7 days | 4 | (5.6) |  | 0 | (0.0) |  | 0 | (0.0) |  |  |
| 30-day readmission‡ | 1 | (1.4) |  | 0 | (0.0) |  | 0 | (0.0) |  | 0.62 |
|  | Median | IQR |  | Median | IQR |  | Median | IQR |  |  |
| Duration of anesthesia, minutes | 308 | (266–340) |  | 343 | (275–393) |  | 342 | (294–418) |  | 0.008 |
| Length of stay, days | 10 | (8–13) |  | 10 | (8–12) |  | 9 | (7–11) |  | 0.30 |
| Total hospitalization cost, USD | 16,884 | (15,573–19,028) |  | 17,467 | (16,189–18,682) |  | 16,456 | (15,389–18,604) |  | 0.19 |

Data are presented as n (%) or median (interquartile range).

*The P-value was calculated to compare four groups using Fisher’s exact test for binary and categorical variables, and the Kruskal–Wallis test for continuous variables.

^†^The items “urinary tract infection” and “acute renal failure” were omitted from in-hospital morbidities because no patients presented with these items.

^‡^Readmission due to congenital pulmonary airway malformation

Abbreviations: ICU, intensive care unit; IQR, interquartile range; USD, United States dollars.

**Supplemental Table 3. Multivariable regression analysis for duration of anesthesia among patients who did not need respiratory support during the neonatal period in the infant group**

|  |  | Coef |  | 95% CI |  | P-value |
| --- | --- | --- | --- | --- | --- | --- |
| Sex |  |  |  |  |  |  |
| Male |  | −2.4 |  | −56.6 to 51.9 |  | 0.93 |
| Female |  | Ref |  |  |  |  |
| Age at surgery |  |  |  |  |  |  |
| 28 days–5 months |  | Ref |  |  |  |  |
| 6–12 months |  | 13.4 |  | −69.3 to 96.1 |  | 0.75 |
| >12 months |  | 43.0 |  | −67.7 to 153.7 |  | 0.44 |
| Body weight at admission, kg |  | −2.4 |  | −20.8 to 15.9 |  | 0.79 |
| Congenital malformation |  |  |  |  |  |  |
| Yes |  | −30.1 |  | −100.5 to 40.3 |  | 0.40 |
| No |  | Ref |  |  |  |  |
| Emergency admission |  |  |  |  |  |  |
| Yes |  | 0.03 |  | −113.7 to 113.8 |  | 0.99 |
| No |  | Ref |  |  |  |  |
| Hospital volume, per year |  |  |  |  |  |  |
| ≤1.6 |  | Ref |  |  |  |  |
| >1.6 |  | −12.3 |  | −68.2 to 43.6 |  | 0.66 |
| Preoperative admission* |  |  |  |  |  |  |
| Yes |  | 177.9 |  | 75.0 to 280.9 |  | 0.001 |
| No |  | Ref |  |  |  |  |
| Thoracoscopic surgery |  |  |  |  |  |  |
| Yes |  | 37.1 |  | −15.6 to 89.8 |  | 0.16 |
| No |  | Ref |  |  |  |  |

*Preoperative admission due to bacterial pneumonia

Abbreviations: Coef, Coefficient; CI, confidence interval; Ref, reference.

**Supplemental Table 4. Multivariable regression analysis for length of stay among patients who did not need respiratory support during the neonatal period in the infant group**

|  |  | Coef |  | 95% CI |  | P-value |
| --- | --- | --- | --- | --- | --- | --- |
| Sex |  |  |  |  |  |  |
| Male |  | −14.9 |  | −32.6 to 2.9 |  | 0.10 |
| Female |  | Ref |  |  |  |  |
| Age at surgery |  |  |  |  |  |  |
| 28 days–5 months |  | Ref |  |  |  |  |
| 6–12 months |  | 3.5 |  | −23.5 to 30.6 |  | 0.80 |
| >12 months |  | 8.9 |  | −27.3 to 45.1 |  | 0.62 |
| Body weight at admission, kg |  | 0.2 |  | −5.8 to 6.2 |  | 0.95 |
| Congenital malformation |  |  |  |  |  |  |
| Yes |  | 20.4 |  | −2.6 to 43.4 |  | 0.081 |
| No |  | Ref |  |  |  |  |
| Emergency admission |  |  |  |  |  |  |
| Yes |  | 128.9 |  | 91.7 to 166.1 |  | <0.001 |
| No |  | Ref |  |  |  |  |
| Hospital volume, per year |  |  |  |  |  |  |
| ≤1.6 |  | Ref |  |  |  |  |
| >1.6 |  | 0.9 |  | −17.4 to 19.2 |  | 0.92 |
| Preoperative admission* |  |  |  |  |  |  |
| Yes |  | 1.2 |  | −32.5 to 34.9 |  | 0.94 |
| No |  | Ref |  |  |  |  |
| Thoracoscopic surgery |  |  |  |  |  |  |
| Yes |  | −11.3 |  | −28.5 to 6.0 |  | 0.20 |
| No |  | Ref |  |  |  |  |

Abbreviations: Coef, Coefficient; CI, confidence interval; Ref, reference

*Preoperative admission due to bacterial pneumonia

**Supplemental Table 5. Demographic and clinical characteristics of patients who underwent thoracoscopic surgery in the infant group, categorized by age**

|  | Infant group | | | | | | | |  |  |
| --- | --- | --- | --- | --- | --- | --- | --- | --- | --- | --- |
|  | 28days–5months | |  | 6–12months | |  | >12months | |  |  |
|  | n=27 | |  | n=27 | |  | n=50 | |  | P-value* |
| Male sex | 16 | (59) |  | 13 | (48) |  | 32 | (64) |  | 0.42 |
| Age at surgery, days | 125 | (47–152) |  | 271 | (197–309) |  | 593 | (469–1,008) |  | <0.001 |
| Body weight at admission, kg | 5.6 | (3.1–6.6) |  | 8.1 | (7.2–8.5) |  | 10.2 | (8.8–11.4) |  | <0.001 |
| Premature birth (< 37 weeks) | 2 | (7.4) |  | 0 | (0.0) |  | 6 | (12) |  | 0.17 |
| Congenital malformation | 3 | (11) |  | 1 | (3.7) |  | 1 | (2.0) |  | 0.24 |
| Respiratory support† | 6 | (22) |  | 6 | (22) |  | 11 | (22) |  | 0.99 |
| Oxygen | 3 | (11) |  | 5 | (19) |  | 6 | (12) |  | 0.75 |
| Nasal high flow | 0 | (0.0) |  | 0 | (0.0) |  | 0 | (0.0) |  | N.A. |
| IPPV | 0 | (0.0) |  | 0 | (0.0) |  | 0 | (0.0) |  | N.A. |
| Mechanical ventilation | 3 | (11) |  | 1 | (3.7) |  | 6 | (12) |  | 0.57 |
| Emergency admission | 6 | (22) |  | 1 | (3.7) |  | 0 | (0.0) |  | 0.001 |
| Ambulance use | 0 | (0.0) |  | 0 | (0.0) |  | 0 | (0.0) |  | N.A. |
| Hospital volume, per year |  |  |  |  |  |  |  |  |  | 0.001 |
| ≤1.6 | 4 | (15) |  | 12 | (44) |  | 28 | (56) |  |  |
| >1.6 | 23 | (85) |  | 15 | (56) |  | 22 | (44) |  |  |
| Preoperative admission‡ | 1 | (3.7) |  | 3 | (11) |  | 4 | (8.0) |  | 0.58 |
| Number of times admitted before surgery‡ |  |  |  |  |  |  |  |  |  | 0.23 |
| '1 | 1 | (3.7) |  | 1 | (3.7) |  | 4 | (8.0) |  |  |
| '2 | 0 | (0.0) |  | 2 | (7.4) |  | 0 | (0.0) |  |  |
| Surgical procedure |  |  |  |  |  |  |  |  |  | 0.69 |
| Thoracoscopic wedge resection | 3 | (11) |  | 2 | (7.4) |  | 7 | (14) |  |  |
| Thoracoscopic lobectomy | 24 | (89) |  | 25 | (93) |  | 43 | (86) |  |  |

Data are presented as n (%) or median (interquartile range).

* The P-value was calculated to compare four groups using Fisher’s exact test for binary and categorical variables, and the Kruskal–Wallis test for continuous variables.

^†^ Respiratory support within the first 2 days after admission during the neonatal period.

^‡^ Preoperative admission due to bacterial pneumonia.

Abbreviation: IPPV, intermittent positive pressure ventilation

**Supplemental Table 6. Comparisons of outcomes of patients who underwent thoracoscopic surgery in the infant group, categorized by age**

|  | Infant group | | | | | | | |  |  |
| --- | --- | --- | --- | --- | --- | --- | --- | --- | --- | --- |
|  | 28 days–5 months | |  | 6–12 months | |  | >12 months | |  |  |
|  | 27 | |  | 27 | |  | 50 | |  | P-value |
| In-hospital mortality | 0 | (0.0) |  | 0 | (0.0) |  | 0 | (0.0) |  | N.A. |
| In-hospital morbidity† | 4 | (15) |  | 3 | (11) |  | 2 | (4.0) |  | 0.25 |
| Surgical site infection | 0 | (0.0) |  | 1 | (3.7) |  | 0 | (0.0) |  | 0.52 |
| Postoperative bleeding | 1 | (3.7) |  | 1 | (3.7) |  | 1 | (2.0) |  | 0.99 |
| Respiratory complication | 3 | (11.1) |  | 2 | (7.4) |  | 1 | (2.0) |  | 0.16 |
| Reoperation | 0 | (0.0) |  | 1 | (3.7) |  | 1 | (2.0) |  | 0.99 |
| Blood transfusion use | 5 | (19) |  | 2 | (7) |  | 2 | (4.0) |  | 0.08 |
| Catecholamine use | 4 | (15) |  | 2 | (7.4) |  | 6 | (12) |  | 0.68 |
| ICU admission | 21 | (78) |  | 16 | (59) |  | 28 | (56) |  | 0.16 |
| After surgery | 18 | (67) |  | 16 | (59) |  | 28 | (56) |  | 0.69 |
| Duration of ICU stay |  |  |  |  |  |  |  |  |  | 0.001 |
| ≤2 days | 16 | (59) |  | 14 | (52) |  | 28 | (56) |  |  |
| 3–7 days | 0 | (0.0) |  | 2 | (7.4) |  | 3 | (6.0) |  |  |
| >7 days | 5 | (19) |  | 0 | (0.0) |  | 0 | (0.0) |  |  |
| 30-day readmission‡ | 0 | (0.0) |  | 0 | (0.0) |  | 0 | (0.0) |  | N.A. |
|  | Median | IQR |  | Median | IQR |  | Median | IQR |  |  |
| Duration of anesthesia, minutes | 317 | (254–351) |  | 345 | (271–394) |  | 394 | (306–456) |  | 0.011 |
| Length of stay, days | 10 | (7–43) |  | 8 | (7–12) |  | 9 | (7–11) |  | 0.18 |
| Total hospitalization cost, USD | 17,889 | (15,796–28,146) |  | 17,933 | (15,697–20,373) |  | 17,470 | (15,474–19,877) |  | 0.37 |

Data are presented as n (%) or median (interquartile range).

*The P-value was calculated to compare four groups using Fisher’s exact test for binary and categorical variables, and the Kruskal–Wallis test for continuous variables.

^†^The items “pulmonary embolism”, “cardiac events”, “sepsis”, “urinary tract infection”, and “acute renal failure” were omitted from in-hospital morbidities because no patients presented with these items.

^‡^Readmission due to congenital pulmonary airway malformation

Abbreviations: ICU, intensive care unit; IQR, interquartile range; USD, United States dollars.

**Supplemental Table 7. Multivariable regression analysis for duration of anesthesia among patients who underwent thoracoscopic surgery in the infant group**

|  |  | Coef |  | 95% CI |  | P-value |
| --- | --- | --- | --- | --- | --- | --- |
| Sex |  |  |  |  |  |  |
| Male |  | 27.5 |  | −18.7 to 73.8 |  | 0.24 |
| Female |  | Ref |  |  |  |  |
| Age at surgery |  |  |  |  |  |  |
| 28 days–5 months |  | Ref |  |  |  |  |
| 6–12 months |  | 39.7 |  | −30.5 to 110.0 |  | 0.26 |
| >12 months |  | 117.5 |  | 39.2 to 195.7 |  | 0.004 |
| Body weight at admission, kg |  | −6.5 |  | −15.7 to 2.7 |  | 0.17 |
| Congenital malformation |  |  |  |  |  |  |
| Yes |  | −16.2 |  | −123.7 to 91.3 |  | 0.77 |
| No |  | Ref |  |  |  |  |
| Emergency admission |  |  |  |  |  |  |
| Yes |  | 0.4 |  | −97.0 to 97.9 |  | 0.99 |
| No |  | Ref |  |  |  |  |
| Hospital volume, per year |  |  |  |  |  |  |
| ≤1.6 |  | Ref |  |  |  |  |
| >1.6 |  | 24.6 |  | −22.8 to 72.0 |  | 0.31 |
| Preoperative admission* |  |  |  |  |  |  |
| Yes |  | 68.5 |  | −15.4 to 152.3 |  | 0.11 |
| No |  | Ref |  |  |  |  |

*Preoperative admission due to bacterial pneumonia

Abbreviations: Coef, Coefficient; CI, confidence interval; Ref, reference.

**Supplemental Table 8. Multivariable regression analysis for length of stay among patients who underwent thoracoscopic surgery in the infant group**

|  |  | Coef |  | 95% CI |  | P-value |
| --- | --- | --- | --- | --- | --- | --- |
| Sex |  |  |  |  |  |  |
| Male |  | 2.1 |  | −0.2 to 4.5 |  | 0.075 |
| Female |  | Ref |  |  |  |  |
| Age at surgery |  |  |  |  |  |  |
| 28 days–5 months |  | Ref |  |  |  |  |
| 6–12 months |  | 0.7 |  | −2.8 to 4.2 |  | 0.69 |
| >12 months |  | 1.0 |  | −2.9 to 5.0 |  | 0.60 |
| Body weight at admission, kg |  | -0.3 |  | −0.8 to 0.2 |  | 0.21 |
| Congenital malformation |  |  |  |  |  |  |
| Yes |  | 0.2 |  | −5.3 to 5.6 |  | 0.95 |
| No |  | Ref |  |  |  |  |
| Emergency admission |  |  |  |  |  |  |
| Yes |  | 16.3 |  | 11.3 to 21.2 |  | <0.001 |
| No |  | Ref |  |  |  |  |
| Hospital volume, per year |  |  |  |  |  |  |
| ≤1.6 |  | Ref |  |  |  |  |
| >1.6 |  | 0.6 |  | −1.8 to 3.0 |  | 0.63 |
| Preoperative admission* |  |  |  |  |  |  |
| Yes |  | −0.7 |  | −5.0 to 3.5 |  | 0.73 |
| No |  | Ref |  |  |  |  |

Abbreviations: Coef, Coefficient; CI, confidence interval; Ref, reference

*Preoperative admission due to bacterial pneumonia
